# Alpha-Ketoglutarate: A Metabolic Regulator of Cellular Homeostasis and Pathophysiology

**DOI:** 10.3390/biomedicines14040836

**Published:** 2026-04-07

**Authors:** Vinay Devulapalli, Akash Sathiyamurthi, Surabhi Gautam, Pallavi Bhattaram

**Affiliations:** Department of Orthopaedics, Emory University School of Medicine, 615 Michael Street, Atlanta, GA 30322, USA; vinaydevulapalli1@gmail.com (V.D.);

**Keywords:** alpha-ketoglutarate, inflammation, TCA cycle, macrophage, epigenetics

## Abstract

Alpha-Ketoglutarate (AKG), a central intermediate of the tricarboxylic acid cycle, is a crucial metabolic and signaling molecule that connects mitochondrial function with cellular homeostasis, immunological modulation, epigenetic remodeling, and lifespan. While mitochondrial AKG maintains energy metabolism, the nuclear AKG pool influences chromatin remodeling through DNA and histone modifications, which together control hypoxia responses and shape gene expression patterns. This dual role demonstrates AKG’s significance in mediating metabolic state, gene expression, and long-term cellular adaptability. AKG modulates immunological responses, reduces reactive oxygen species (ROS), promotes the polarization of anti-inflammatory macrophages, and suppresses nuclear factor kappa B (NF-κB) activation, thereby reducing chronic inflammatory processes. AKG restricts pro-inflammatory cytokine production, increases extracellular matrix synthesis, and reduces cartilage degradation in arthritic models, suggesting potential therapeutic benefits in autoimmune diseases and joint degeneration. Additionally, AKG affects lifespan in several model organisms, where supplementation enhances metabolic resilience, lowers age-related inflammation, modifies mTOR signaling, and preserves youthful epigenetic profiles. Additionally, because endogenous AKG levels decrease with age, oral supplementation of AKG, especially with calcium and arginine, has drawn attention to its potential benefits in longevity and metabolic health. Thus, AKG is versatile and has encouraging therapeutic promise for cancer, aging, and inflammatory illnesses. However, a lack of human clinical evidence prompts further research to determine ideal dosage, tissue selectivity, and long-term safety. The goal of this review is to critically examine the current mechanistic knowledge related to AKG biosynthesis and breakdown and its future implications in maintaining cellular homeostasis and controlling chronic inflammation.

## 1. Introduction

Cellular metabolism is intrinsically linked to immune modulation, which determines how cells detect, respond to, and recover from inflammatory stress [1]. Among the primary metabolic pathways, the tricarboxylic acid (TCA) or Krebs cycle plays a critical role in energy production, redox homeostasis, and biosynthetic flux. Beyond its traditional role in producing ATP and reducing equivalents, the TCA cycle has emerged as an important center in immunometabolism, where metabolites act as signaling molecules, influencing epigenetics, gene expression, cellular differentiation, and cytokine release [2]. Dysregulation of TCA cycle enzymes disrupts the balance between pro- and anti-inflammatory responses, contributing to the development of chronic inflammatory and autoimmune disorders [3].

Alpha-ketoglutarate (AKG) is a key metabolite in the TCA cycle required for cellular energy synthesis [4]. AKG also contributes to nitrogen metabolism by transamination of glutamate, a precursor for amino acids such as gamma-aminobutyric acid (GABA), glutathione (GSH), and amino acids such as aspartate, glutamine, leucine, and proline [5]. These amino acids are essential for protein synthesis, neurotransmission, and detoxification, which aids in cellular repair and function [4,6]. AKG plays a role in the decomposition of H_2_O_2_, scavenges cellular ammonia and antagonizes lipid peroxidation. In the nucleus, AKG serves as the metabolite for DNA and histone-modifying enzymes, enabling it to control the cells’ epigenetic signature. These biological properties demonstrate the significant contributions of AKG to cellular energy production, oxidative stress prevention, amino acid synthesis, epigenetic regulation, and nitrogen homeostasis. The bioavailability of this multifunctional cell metabolite depends on its dynamic production and breakdown according to cellular need. In the TCA cycle, AKG is mainly produced from isocitrate through oxidative decarboxylation catalyzed by isocitrate dehydrogenase (IDH) [7]. It can also be synthesized anaerobically from glutamate by oxidative deamination with glutamate dehydrogenase or as a byproduct of pyridoxal phosphate-dependent transamination events in which glutamate is a common amino source [8].

Studies from a variety of organ systems, including the musculoskeletal, renal, gastrointestinal, cardiovascular, nervous, and immune system, suggest that AKG dietary supplementation may have many beneficial health effects [5,9,10,11,12,13]. Research so far indicates that AKG supplementation may promote tissue integrity, maintain metabolic equilibrium during homeostasis, and have potential therapeutic applications in a variety of immune and metabolism-related pathological conditions. Some of the reported activities of AKG include promoting bone matrix formation, reducing muscle degradation, and increasing muscle strength and endurance [5,14]. AKG, also called the immune nutrition factor, plays a crucial part in overall immune metabolism. It affects macrophage polarization, altering the balance of pro-inflammatory to anti-inflammatory phenotype during bone repair and regeneration [15]. It was suggested that AKG supplementation reduces inflammation by stabilizing lipopolysaccharide-mediated production of tumor necrosis factor alpha (TNFα) and insulin-like growth factor 1 (IGF-1) in liver Kupffer macrophage cells [16]. AKG suppressed phosphorylated-Akt (pAkt)and pP65 (canonical NF-kB) signaling in the lungs, reducing platelet and leukocyte infiltration and inflammatory cytokine buildup [17]. AKG was also reported to work via inhibiting mammalian target of rapamycin (mTOR) and ATP synthase, modifying DNA and histone demethylation, and lowering Reactive Oxygen Species (ROS) production [18]. Based on this premise, this review sought to analyze the role and mechanisms of AKG and to understand the pathways that AKG and the enzymes involved in its biosynthesis could impact inflammation. Further, we discuss how changes in TCA cycle flux affect immune cell function, emphasize AKG’s role as an epigenetic and redox regulator, and assess the translational potential of targeting these metabolic pathways in inflammatory disorders.

## 2. Importance of AKG Biosynthesis Enzymes in Cellular Physiology

**Figure 1:**
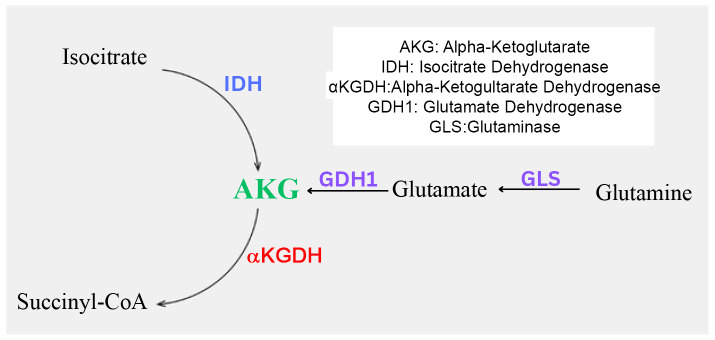
Schematic showing the enzymatic pathways involved in the biosynthesis and breakdown of AKG. Tricarboxylic acid cycle: The conversion of Isocitrate into AKG through the Isocitrate Dehydrogenase Enzyme and the conversion of AKG to Succinyl-CoA through AKG Dehydrogenase. Glutamine metabolism: The conversion of Glutamine into Glutamate through Glutaminase and the conversion of Glutamate into AKG through Glutamate Dehydrogenase.

Two major cellular pathways result in AKG biosynthesis—the TCA Cycle and the Glutaminase Pathway 1 (Figure 1). Within these two pathways, three enzymes primarily facilitate AKG biosynthesis. These include Isocitrate Dehydrogenase (IDH), Glutamate Dehydrogenase (GDH1), and Glutaminase (GLS). IDH is a TCA cycle enzyme that converts Isocitrate to AKG through the process of oxidative decarboxylation as part of the TCA cycle [19]. Outside of the TCA cycle, select amino acids can be converted to AKG through alternate pathways, via the glutaminase pathway. This conversion begins with the deamination of glutamine to glutamate using the enzyme GLS. Following this, GDH1 converts glutamate into AKG via a transaminase reaction [20]. On the other hand, AKG is constantly metabolized in the TCA cycle through the enzyme Alpha-ketoglutarate Dehydrogenase (aKGDH); aKGDH converts AKG to succinyl-CoA [21]. It has been reported that the higher the activity of aKGDH and the higher the metabolic degradation of AKG, the stronger the oxidative stress build-up within cells [21]. In the following paragraphs, we will briefly discuss the functions of these critical enzymes that maintain AKG bioavailability.

Isocitrate dehydrogenases (IDHs) are a class of key proteins in energy metabolism, including IDH1, IDH2, and nicotinamide adenine dinucleotide-dependent IDH3. These enzymes are involved in the oxidative decarboxylation of isocitrate to yield α-ketoglutarate (AKG), which is the rate-limiting step of the TCA cycle [22]. IDH2 and IDH3 are the main drivers of the TCA cycle as they are localized to the mitochondrial compartment [23]. IDH1 and IDH2 have been additionally reported to translocate from the cytosol to the nucleus, where they regulate histone and DNA modifications [24]. Under normoxia, IDHs participate in glucose-derived pyruvate through the citrate shuttle and ATP citrate lyase. However, under conditions of hypoxia or mitochondrial dysfunction, they mediate reductive carboxylation of glutamine-derived α-KG to produce acetyl CoA toward fatty acid biosynthesis, thereby supporting tumor growth and progression. AKG biosynthesis by IDHs is also necessary to maintain an adequate pool of potent antioxidant factors such as reduced glutathione (GSH) and peroxiredoxin. Thus, the production of AKG by IDH1, IDH2, and IDH3 in various cell compartments plays important roles in epigenetic and metabolic and pathological activities, including glucose sensing, glutamine metabolism, lipogenesis, and regulation of cellular redox status. Moreover, mutations in the coding domain for IDH1 and IDH2 were reported to cause the production of the oncometabolite 2-hydroxyglutarate (2-HG) instead of AKG, resulting in abnormal cell proliferation in cancers like glioma and acute myeloid leukemia (AML) [24]. These mutations also resulted in epigenetic deregulation, as 2-HG can prevent histone demethylation by inhibiting AKG-dependent demethylases [25].

The glutaminase pathway comprises two crucial enzymes, GDH and GLS, which are involved in AKG biosynthesis (Figure 1). GDH is the second enzyme of this pathway. It is involved in the direct conversion of glutamate to AKG via oxidative deamination. Furthermore, it uses NAD+ and NADP+ as cofactors in this process [26]. There are two isoforms of GDH, GDH1 and GDH2. However, in this review, we will focus only on GDH1 due to its presence all over the body, and the role of GDH2 is incompletely understood. Guanosine triphosphate (GTP) inhibits GDH1 and is mainly found in the mitochondrial matrix, like IDH2 and IDH3 [26]. GDH1 plays an important role in cell proliferation, especially in glucose-deprived conditions [27]. GLS is an enzyme that acts as the first step of the glutaminase 1 pathway and is located in the mitochondria [28]. Through its ability to transform glutamine into many different products like AKG and Glutamate, GLS is observed to promote cell proliferation and cell survival in human endothelial cells [28]. This means that inhibiting this enzyme can make the cell more susceptible to oxidative stress, as less AKG is produced [28]. GLS also plays an important role in the cell cycle, where GLS1 allows for the progression of cells into the S phase for DNA Synthesis [28]. One study, which aimed to understand the effects of GLS Inhibition in the TCA cycle, found that the resulting deprivation of glutamine showed decreased cell growth. However, administering a variant of dimethyl AKG and asparagine, both of which are downstream products of this pathway, allowed for the restoration of cell growth [28].

Alpha-ketoglutarate dehydrogenase complex (aKGDH) catalyzes the fourth step in the TCA cycle that converts AKG to succinyl-CoA and thereby determines the cellular availability of AKG. This complex consists of three main components: E1 (alpha-ketoglutarate dehydrogenase), E2 (dihydrolipoamide succinyl transferase), and E3 (dihydrolipoamide dehydrogenase). The E1 subunit initiates the process by accepting the alpha-ketoglutarate substrate, while the E2 subunit forms the core structure of the complex. The E3 subunit regenerates the oxidized form of lipoamide required for the E2 component. aKGDH’s role in managing oxidative stress is a topic that has seen varying observations. On one hand, aKGDH plays a role in mitochondrial antioxidant mechanisms as it can produce energy to stop oxidative stress. On the other hand, aKGDH can produce ROS, like superoxide and hydrogen peroxide, in response to the NADH/NAD+ ratio within a cell [29]. With these two facts at hand, aKGDH activity must be regulated under tight control, as over- or underactivation can have devastating effects within a cell. The therapeutic importance of AKDGH is based on studies exploring the pathogenesis of various cancer cell lines. In AML lymphocyte lines, it was shown that the inhibition of 2-AKG dehydrogenase, the E1 subunit of the aKGDH complex, impaired AML progression and drove differentiation. Mechanistically, hindrance of AKG flux through the tricarboxylic acid (TCA) cycle resulted in rapid exhaustion of aspartate pools and blockade of de novo nucleotide biosynthesis, whereas cellular bioenergetics was largely preserved. Furthermore, aKGDH inhibition led to increased AKG levels, which affected the biosynthesis of other critical amino acids [30]. Interestingly, recent reports also show that the aKGDH complex can translocate into the nucleus in the U251 human brain cancer cell line, facilitating the enzymatic conversion of nuclear AKG to succinyl-CoA, which is subsequently utilized for histone lysine succinylation [31]. Taken together, the metabolism of AKG by the AKDGH complex has versatile and context-dependent roles, warranting further investigations to decipher its homeostatic and pathological significance.

## 3. Molecular Mechanism Underlying AKG Biological Activity

### 3.1. Mitochondrial Function and Cell Signaling

AKG, a central mitochondrial metabolite of the tricarboxylic acid (TCA) cycle, possesses the potential to integrate cellular bioenergetic, redox, and signaling functions to maintain homeostasis. Beyond its role in ATP production, AKG serves as a major hub for mitochondrial nitrogen and amino acid metabolism through reversible interconversion with glutamate [32]. In proliferating cells, such as immune cells, cancer cells, and stem cells, AKG acts as the coupling metabolite between the TCA cycle and anaplerosis, a metabolic process by which cells replenish intermediates of the TCA cycle using amino acids, nucleotides, and lipids as precursors [33,34]. AKG also plays a critical role in mitochondrial redox regulation; in neurons, AKG attenuates oxidative stress and restores mitochondrial function during H_2_O_2_-induced injury [35], and in chondrocytes, α-KG decreases ROS accumulation and ferroptotic damage under oxidative stress [36]. In cardiomyocytes, AKG supplementation reduces mitochondrial ROS, improves membrane potential, and enhances mitophagy, protecting against oxidative injury [37]. Similarly, in immune cells such as dendritic cells, AKG modulates redox metabolism and antioxidant defenses [38], and in systemic inflammatory models, AKG alleviates mitochondrial oxidative stress in immune tissues such as the spleen [39], further demonstrating its role in redox homeostasis across cell types. Alterations in AKG levels and AKG/succinate ratios have been implicated in aging, cancer, neurodegeneration, and musculoskeletal diseases, affecting neurons, synoviocytes, chondrocytes, osteoblasts, and immune cells [7,40,41], where they contribute to mitochondrial dysfunction, aberrant redox signaling, and pathological cellular remodeling. For example, AKG modulates longevity and metabolic signaling through mTOR/AMPK pathways [42]. Together, these findings establish AKG as a central mitochondrial metabolite whose balanced regulation is critical for cellular and organismal health, while its disruption drives disease-associated metabolic and signaling defects across diverse cell types.

### 3.2. Epigenetic Regulation

Although primarily generated in mitochondria through the tricarboxylic acid (TCA) cycle, AKG is transported to the cytosol and nucleus, where it supports the activity of ten–eleven translocation (TET) DNA demethylases and Jumonji C (JmjC) domain–containing histone demethylases, a mechanism initially characterized in embryonic stem cells and cancer cells [43,44]. In these cell types, adequate nuclear AKG availability promotes DNA and histone demethylation, maintaining transcriptional programs associated with pluripotency, differentiation, and cellular homeostasis [45]. In contrast, reduced AKG levels or increased accumulation of competing TCA metabolites such as succinate and fumarate inhibit AKG–dependent dioxygenase activity, leading to epigenetic repression and aberrant gene expression, as demonstrated in tumor cells and immune cells [46]. The α-ketoglutarate dehydrogenase enzyme complex plays a pivotal role in regulating intracellular AKG and succinyl-CoA levels, with its metabolic control functions originally defined in neuronal and hepatic mitochondria and later extended to proliferating and immune cells [47]. Notably, aKGDH-derived succinyl-CoA serves as a substrate for histone lysine succinylation, a post-translational modification identified in mammalian cell lines and macrophages that alters chromatin structure and transcriptional activity, thereby linking mitochondrial metabolic flux to nuclear epigenetic regulation [48,49]. Dysregulation of aKGDH activity and succinyl-CoA availability can therefore shift the balance between AKG-dependent demethylation and histone succinylation, contributing to altered gene expression programs observed in cancer cells and aging-associated tissues [50].

## 4. Pathophysiological Roles and Therapeutic Applications of AKG

Emerging evidence supports an important role for AKG that links cellular metabolic stress to chronic inflammation. Here we discuss some of the key findings related to chronic inflammatory diseases, including musculoskeletal, neurodegenerative, and autoimmune diseases and cancer, which have been associated with alterations in AKG levels [50,51,52].

One study examined the significance of AKG in osteoarthritis (OA), a chronic joint disease that progressively degenerates the articular cartilage and its surrounding synovial tissues, leading to painful joints and disability [36]. AKG was found to inhibit articular cartilage degeneration by decreasing the transcription of pro-inflammatory cytokines, such as Interleukin-1 beta (IL-1β), alongside inhibiting chondrocyte cell death [36]. In rodent models of OA, AKG promoted cartilage repair by upregulating matrix synthesis genes like Collagen type II alpha 1 (COL2A1), reduced harmful ROS production, and restored mitochondrial health [36]. In addition, AKG mitochondrial quality control via mitophagy to further alleviate oxidative stress in OA joints [36]. Rheumatoid arthritis (RA) is a chronic autoimmune joint disease, where inflammation drives a major portion of the symptoms experienced by patients, specifically the development of synovitis. Macrophages are known to play a major role in regulating synovitis [53]. The M1-like macrophage is considered pro-inflammatory, where its activation causes and sustains inflammation in the synovial and peripheral blood [53,54]. These M1-like macrophages secrete pro-inflammatory cytokines like IL-1, IL-12, and IL-23. M2 macrophages exhibit anti-inflammatory properties and produce anti-inflammatory effects in response to ongoing inflammation [54]. AKG has been found to partake in macrophage polarization, as different signaling cascades can be manipulated to alter the frequencies of M1-like and M2-like macrophages [55]. In particular, AKG has been found to inhibit M1-like activation by suppressing the NF-kB pathway while also promoting the activation and programming of M2-like anti-inflammatory phenotype [54]. By doing so, AKG plays a role in maintaining a higher M2/M1 ratio, reducing inflammation. The AKG/succinate ratio is important in this aspect, as the higher ratios tend to favor M2-like polarization to lower inflammatory levels [55]. Therefore, AKG administration is a promising modality to reduce pro-inflammatory macrophage polarization and achieve an overall balance in the inflammation response of joint cells.

Neurons are highly dependent on intact mitochondrial oxidative metabolism for ATP generation, calcium buffering, and neurotransmitter cycling. In the brain, in addition to a role in TCA cycle-related energy metabolism, AKG is a precursor for both excitatory neurotransmitter glutamate and the inhibitory neurotransmitter γ-aminobutyric acid (GABA) [56]. AKG treatment was also shown to promote synaptic vesicle fusion with the presynaptic membrane via increasing the sensitivity of synaptotagmin 1 (Syt1) to calcium and thereby supporting neurogenesis and cognitive functions [57]. In the case of cerebral ischemia, which causes glucose and oxygen deprivation, higher levels of AKG may inhibit mTOR signaling as well as the inhibition of ATP synthase, which is a direct target of AKG [6,42,58]. Another mechanism through which AKG might exert a role in neurodegeneration is via modulating the expression and activation of Toll-like Receptors (TLR) [59,60]. This notion is based on reports from inflammatory conditions such as lupus, which indicate that AKG induces anti-inflammatory effects by inhibiting TLR signaling, and downstream nuclear factor (NF)-kB signaling activation [61]. However, TLR 2 and 4 signaling pathways could also enhance the phagocytosis of Aβ plaques, providing a protective effect [62,63,64]. Therefore, TLR inhibition in microglial cells by AKG might have negative consequences in neurodegenerative conditions associated with amyloid deposition. Indeed, aKGDH complex deficiency is one of the most consistently reported metabolic defects in neurodegenerative diseases, particularly Alzheimer’s disease (AD), where reduced aKGDH activity has been documented in post-mortem AD brains. Moreover, this correlates with impaired TCA cycle flux, reduced ATP production, and increased oxidative stress, further accelerating neurodegeneration [65,66]. Based on this available information, we posit that the higher AKG levels caused by impaired aKGDH activity may be detrimental in neurodegenerative diseases, whereas higher levels of AKG, under normal aKGDH activity, might be neuroprotective.

Cancer cells are known to undergo massive metabolic reprogramming, which results in redirecting the energy cycle metabolites, including the tricarboxylic acid (TCA) cycle intermediates, towards biosynthesis to sustain rapid proliferation. Moreover, mutations in TCA cycle enzymes are known to accumulate in cancer cells, leading to the production of oncometabolites that can alter gene expression through epigenetic modifications. Some common oncometabolites include succinate, fumarate, itaconate, and 2-HG, all of which can alter epigenetic modifications, such as preventing histone demethylation [25,67]. Below, we discuss literature reports from the cancer field that report context-dependent activities of AKG as a tumor progressor or as an anti-cancer metabolite. Prior studies indicate that AKG can act as a competitive inhibitor of oncometabolites, and restoring the AKG/oncometabolite ratio can inhibit cancer cell growth. As previously discussed, mutations that lead to 2-HG accumulation can be reversed by increasing AKG amounts [68,69]. Similarly, mutations leading to the disruption of mitochondrial complex I activity were shown to increase the AKG to Succinate ratio, leading to HIF-1α destabilization and reduced tumor growth both in vitro and in vivo [70,71]. Accumulation of AKG due to aKGDH inactivation was also shown to result in the increased activity of DNA demethylases TET1 and TET3 in breast cancer cells, inhibiting their cell migration and epithelial–mesenchymal transition [72]. In addition, an increase in the intracellular AKG levels was shown to stimulate histone demethylation to induce breast cancer cell senescence [73], as well as overcoming the apoptosis resistance of colon cancer cells [74], whereas in human liver and lung cancer cells, AKG deficiency was shown to induce disulfide stress via the inhibition of adenosine 5′-monophosphate-activated protein kinase (AMPK), a master regulator of energy metabolism [75]. In the context of renal cell carcinoma, AKG was shown to promote cancer cell apoptosis by inhibiting the PI3K/AKT/mTOR pathways [76].

While we have seen how AKG can prevent the detrimental effects of cancer, it too can affect cancer growth. Tumor cells can absorb a high level of glutamine and therefore have a high level of AKG, which fuels their mitochondrial function, providing tumor cells with large amounts of energy for growth [67]. Continuing in observations that AKG may contribute to cancer cell growth, one study observed that knocking down GDH1 to cause AKG deficiency caused reduced cancer cell growth [77]. This is because of the stressful conditions that AKG insufficiency may put cancer cells under energy stress and glucose starvation [77]. AKG can also allow prostate tumor cells to survive in a nutrient-poor microenvironment, promoting their metastasis and growth, mainly via modulating the mTOR signaling pathway to enhance protein synthesis and support survival. Overall, existing literature has shown conflicting and context-dependent effects of AKG on cancer [78]. In addition to these findings in cancer, it was also reported that AKG treatment was protective against cisplatin-induced kidney injury, mainly by upregulating mitophagy-related genes, including PINK1 and MNF1 [79]. Taken together, these data highlight the complexity of molecular mechanisms explaining the role of AKG on oncometabolites, the mTOR signaling pathway, and epigenetic regulation of cancer cells. The data also indicates that additional factors such as hypoxia, inflammation, oxidative stress and oncogenic mutations of each cancer cell type will ultimately determine the overall effect of AKG treatment on cell physiology. This creates a possible need for further research to understand if we can view AKG as a cancer therapeutic.

### Dietary Supplementation and Potential Therapeutic Applications

AKG has been shown to have a positive association with lifespan, and there have been many studies focused on this association [42,80,81,82]. One study illustrated that within mouse models, the inclusion of diet-induced AKG supplementation increased Interleukin 10 (IL-10) expression, which is linked to increased anti-inflammatory gene expression [81]. This study also reported that the inhibition of markers, senescence-associated secretory phenotype (SASP), including the interleukins IL1-beta, IL6, CCL2, and MMP3, without an associated change in expression of beta-galactosidase and p21 levels, suggesting that AKG may not directly interfere with the formation of senescent cells, but rather influence the secretory phenotype of the senescent cells. Furthermore, these animals also experienced decreased expression of age-dependent phenotypes such as fur discoloration and dermatitis, as well as improvement in gait and decreased exhaustion. Overall, this study highlights a broader view of AKG supplementation, where long-term aging shows improvement compared to untreated mice [81]. As aging and its associated diseases are closely related to inflammation, this study allows for a different perspective on AKG’s relation to inflammation. On a mechanistic level, AKG also has the capacity to regulate energy production and efficiency due to its regulatory role in ATP synthesis [54]. AKG’s ability to regulate macrophage polarization through M1 and M2 macrophages has been speculated to contribute to the extended lifespan properties, but more research is needed [54]. Another human study recently showed that higher levels of succinyl CoA, a downstream metabolic product of AKG, are associated with longer healthy lifespan in humans, where increased histone lysine succinylation and epigenetic regulation were suggested as the underlying mechanisms [83]. Aging can also be modulated by the mTOR pathway. The mTOR pathway has strong connections to not only aging but also immune system response, autophagy, and mitochondrial function [84]. It has been observed that genetic or treatment-driven inhibition of the mTORC1 kinase can increase lifespan for Eukaryotic organisms [85]. Studies have shown that dietary AKG has been observed to inhibit the mTOR pathway and therefore increase the lifespan of nematodes, but the specific mechanism is unclear [80]. A similar observation was found later in the article on Drosophila [80]. There have been many longevity-focused studies involving different models such as Caenorhabditis elegans, flies, and humans. These studies observed AKG’s impact on aging through wide-ranging effects like regulation of ATP Synthase, maintaining oxidative balance, and decreasing inflammation. AKG also possesses pro-apoptotic and pro-autophagy effects, which allow the body to undergo efficient homeostatic maintenance and cellular quality during aging [76,85].

Supplemental AKG is often provided in the form of ornithine-alpha-ketoglutarate (OKG) [86,87,88]. It is not made naturally but is simply the combination of L-ornithine and AKG. OKG, when used as a food supplement, caused an improvement in the inflammatory status of the intestines of humans and pigs [89]. OKG is also a precursor to glutamine; henceforth, OKG has been illustrated to improve glutamine levels in the blood and improve immune function [89]. With this, we can see how OKG can be tested as a method of supplementing the Glutaminase pathway, increasing AKG levels in the blood, and decreasing inflammation. Another study conducted on pigs infected with enterotoxigenic Escherichia coli (ETEC), a digestive pathogen, found that supplementation of ETEC pigs with OKG had reduced expression of pro-inflammatory marker IL-6 and increased expression of anti-inflammatory marker IL-10 [89]. Now, looking at the effects of dietary AKG, one study examined the effects of dietary AKG on piglets and found that AKG supplementation assisted bone growth and health heavily. This is achieved through AKG’s improvement of piglets’ ileum and digestive tract, allowing them improved digestibility and utilization of calcium and phosphorus for bone growth [90]. As summarized in Table 1, other frequently utilized forms of AKG, which have been extensively used in various animal model research, include sodium-AKG, calcium AKG, AKG-nanoparticle formulations, and cell-permeable dimethyl AKG. However, it is important to note that prior studies have not adequately considered the chemical form of AKG and its specific biological effects. This is particularly important because AKG analogues are known to be rapidly hydrolyzed extracellularly and may enter cells as different chemical forms [91], thereby inducing direct AKG-dependent, but also analogue-dependent effects on the metabolism of various cells. Only a limited number of studies have measured the intracellular levels of AKG or its analogues in the cytoplasm or the nuclear compartment post-treatment [77,91], and thus, reliable information on the level of enhancement of AKG bioavailability for each of the formulations is currently unavailable. A neck-to-neck comparison of the potential benefits and effectiveness for each of these chemical forms is not yet available and is an important factor to consider when developing AKG as a dietary supplement or therapeutic drug. Moreover, considering the wide-ranging effects of AKG in the body as observed in these studies, further research can build on how dietary supplements of AKG can be a worthwhile way to administer the molecules and test the effects we have observed.

## 5. Conclusions and Future Directions

**Figure 2:**
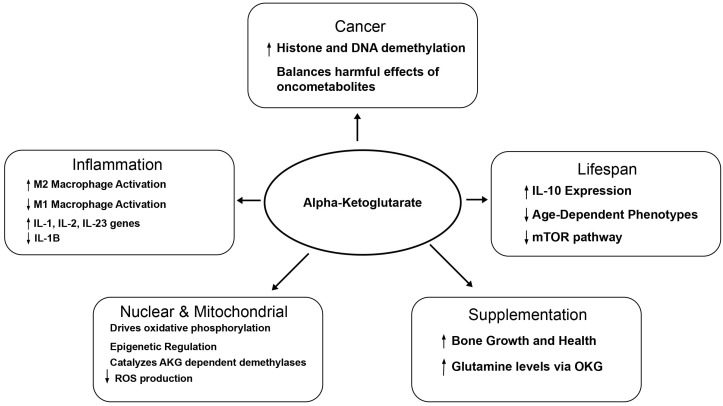
Summary of the biological roles of AKG in health and disease. AKG plays a role in cancer through the balancing of harmful effects of oncometabolites and increasing histone and DNA methylation. AKG is involved in lifespan through its ability to increase IL-10 expression and decrease age-dependent phenotypes and the mTOR pathway. AKG can also increase bone growth and health and increase glutamine levels through OKG. Inflammation is also affected by AKG, in which AKG increases M2 macrophage activation and expression of IL-1, IL-2, and IL-23 genes and decreases M1 macrophage activation and expression of IL-1B. AKG has nuclear and mitochondrial effects through its ability to drive oxidative phosphorylation, an effect on epigenetic regulation, catalyzation of AKG-dependent demethylases, and decreases ROS production.

In summary, the scientific evidence gathered in this review (Figure 2) highlights α-ketoglutarate (AKG) as a central metabolic intermediate of the tricarboxylic acid cycle with critical roles in maintaining mitochondrial health, regulating oxidative stress, shaping chromatin dynamics, and modulating inflammatory gene expression. AKG is also produced via the metabolism of Glutamine. Under most inflammatory conditions, AKG functions as an anti-inflammatory metabolite by inhibiting pro-inflammatory macrophage polarization. However, these effects are highly context dependent, as AKG has also been reported to promote neurodegeneration and support cancer cell activity under specific conditions. For instance, in neurodegenerative diseases, AKG may exert a protective effect by serving as a precursor for neurotransmitters [56] and by inhibiting neuronal inflammation by targeting the NF-kappaB and TLR signaling pathways [59,60,61]. On the other hand, AKG may also inhibit amyloid plaque resolution by glial cells due to the inhibition of TLR signaling [59,60]. Similarly, neurodegenerative conditions associated with aKGDH deficiency may lead to excessive accumulation of AKG in the tissues, causing toxicity [65,66]. At the molecular level, multiple studies in cancer and chronic inflammatory diseases have shown that AKG exerts its effects primarily through regulation of macrophage polarization, histone succinylation, and DNA methylation. We propose that future investigations should focus on delineating the mechanisms by which AKG amplifies histone and DNA demethylation and on leveraging these pathways for the development of cancer therapeutics. Given AKG’s capacity to suppress inflammation, further research is also warranted on macrophage polarization, particularly in relation to the AKG-to-succinate ratio, as this balance may be critical for mitigating inflammation-driven joint diseases and aging. Additionally, this review has discussed multiple AKG formulations used in experimental and clinical contexts, including ornithine AKG, dimethyl AKG, and calcium-AKG, which have demonstrated health-promoting effects but lack consensus regarding their relative efficacy and mechanism of action. Overall, AKG plays a multifaceted role in the metabolism of healthy, as well as diseased cells. Its physiological activity couples cellular metabolism with epigenetic regulation. The beneficial vs. detrimental effects of AKG on cancer, chronic inflammatory and neurodegenerative conditions indicate context-dependent roles. Research so far suggests that the effect of AKG supplementation on a particular biological process is largely influenced by the intracellular localization, level, and activity of the enzymes involved in its biosynthesis and metabolism. Therefore, to fully exploit the therapeutic potential of AKG, future studies need to consider the expression and activity levels of critical AKG-metabolizing enzymes, including α-KGDH, SDH, and GDH1. Collectively, a deeper understanding of AKG-driven metabolic epigenetic crosstalk may further enable the rational design of targeted interventions to modulate inflammation, aging, and disease progression across diverse pathological contexts. Finally, since different formulations of AKG can have unique biological properties, a comprehensive comparative study of each formulation remains an important area for future studies.

## Figures and Tables

**Table 1:** AKG formulations and delivery strategies in human and preclinical studies.

Animal Model	AKG Formulation	Delivery Strategy	Observation
Healthy Humans	Calcium α-Ketoglutarate salt	2 g orally per day 4 to 10 months	Reduction in biological aging based on DNA methylation [82]
Healthy pigs (Duroc × Landrace × Yorkshire)	α-Ketoglutaric acid	500 g/t in the basal diet for 42 days	Reduced diarrhea incidence [92]
Pigs (Duroc × Landrace × Yorkshire) enterotoxic *E. coli*	Ornithine α-Ketoglutarate (OKG)	1% *w*/*w* in basal diet for 3 days	Alleviates inflammation via regulating the Ileal mucosa microbiota [89]
Healthy C57BL6/J mice	Calcium α-Ketoglutarate salt	2% *w*/*w* in regular mouse diet lifelong (18 months)	Extends lifespan by induction of IL10 [81]
Outbred wildtype mice	Sodium α-Ketoglutarate salt	2% *w*/*w* in regular diet, 12-month-old mice for 6 months	Stabilized redox homeostasis and improved arterial elasticity [93]
Collagen-induced arthritis in DBA/1J mice	α-ketoglutarate polymer nanoparticles (paKG NPs)	2 mg paKG NPs from day 21 to 57 post arthritis induction	Mitigated rheumatoid arthritis symptoms in mice and modulated T cell responses [94]
Drosophila	α-ketoglutarate	5 μM in basal diet, 3 times per week, lifelong	Increased lifespan by inhibiting mTOR and activating AMPK [80]
*C. elegans*	8 mM	Every 4 days, lifelong	extends lifespan by inhibiting ATP synthase and mTOR [42]

## Data Availability

No new data were generated for this review article.

## References

[1] Aderinto N., Abdulbasit M.O., Tangmi A.D.E., Okesanya J.O., Mubarak J.M. (2023). Unveiling the growing significance of metabolism in modulating immune cell function: Exploring mechanisms and implications; a review. Ann. Med. Surg..

[2] Liu H., Wang S., Wang J., Guo X., Song Y., Fu K., Gao Z., Liu D., He W., Yang L.L. (2025). Energy metabolism in health and diseases. Signal Transduct. Target. Ther..

[3] Godfrey W.H., Kornberg M.D. (2020). The Role of Metabolic Enzymes in the Regulation of Inflammation. Metabolites.

[4] Wu N., Yang M., Gaur U., Xu H., Yao Y., Li D. (2016). Alpha-Ketoglutarate: Physiological Functions and Applications. Biomol. Ther..

[5] Xu M., Zhang Q., Liu X., Lu L., Li Z. (2024). Impact of Alpha-Ketoglutarate on Skeletal Muscle Health and Exercise Performance: A Narrative Review. Nutrients.

[6] Kostiuchenko O., Lushnikova I., Kowalczyk M., Skibo G. (2022). mTOR/α-ketoglutarate-mediated signaling pathways in the context of brain neurodegeneration and neuroprotection. BBA Adv..

[7] Hansen G.E., Gibson G.E. (2022). The α-Ketoglutarate Dehydrogenase Complex as a Hub of Plasticity in Neurodegeneration and Regeneration. Int. J. Mol. Sci..

[8] Zdzisinska B., Zurek A., Kandefer-Szerszen M. (2017). Alpha-Ketoglutarate as a Molecule with Pleiotropic Activity: Well-Known and Novel Possibilities of Therapeutic Use. Arch. Immunol. Ther. Exp..

[9] Martin R.A., Viggars M.R., Esser K.A. (2023). Metabolism and exercise: The skeletal muscle clock takes centre stage. Nat. Rev. Endocrinol..

[10] Liu H., Xi Q., Tan S., Qu Y., Meng Q., Zhang Y., Cheng Y., Wu G. (2023). The metabolite butyrate produced by gut microbiota inhibits cachexia-associated skeletal muscle atrophy by regulating intestinal barrier function and macrophage polarization. Int. Immunopharmacol..

[11] Wang K., Liu Q., Tang M., Qi G., Qiu C., Huang Y., Yu W., Wang W., Sun H., Ni X. (2023). Chronic kidney disease-induced muscle atrophy: Molecular mechanisms and promising therapies. Biochem. Pharmacol..

[12] Wu D., Fan Z., Li J., Zhang Y., Xu Q., Wang L., Wang L. (2022). Low Protein Diets Supplemented With Alpha-Ketoglutarate Enhance the Growth Performance, Immune Response, and Intestinal Health in Common Carp (*Cyprinus carpio*). Front. Immunol..

[13] Shi Y., Tian M., Zhao X., Tang L., Wang F., Wu H., Liao Q., Ren H., Fu W., Zheng S. (2024). α-Ketoglutarate promotes cardiomyocyte proliferation and heart regeneration after myocardial infarction. Nat. Cardiovasc. Res..

[14] Cai X., Yuan Y., Liao Z., Xing K., Zhu C., Xu Y., Yu L., Wang L., Wang S., Zhu X. (2018). α-Ketoglutarate prevents skeletal muscle protein degradation and muscle atrophy through PHD3/ADRB2 pathway. FASEB J..

[15] Iniguez A.B., Du M., Zhu M.J. (2024). α-Ketoglutarate for Preventing and Managing Intestinal Epithelial Dysfunction. Adv. Nutr..

[16] Li Y., Kuang H., Fan G., Yang X. (2025). Alpha Ketoglutaric Acid attenuates LPS Induced Inflammatory Response by Inhibiting the PKCε/MAPK/P65 Signaling Pathway and Inhibit Oxidative Stress in Kupffer Cells. Inflammation.

[17] Agarwal S., Ghosh R., Verma G., Khadgawat R., Guchhait P. (2023). Alpha-ketoglutarate supplementation reduces inflammation and thrombosis in type 2 diabetes by suppressing leukocyte and platelet activation. Clin. Exp. Immunol..

[18] Naeini S.H., Mavaddatiyan L., Kalkhoran Z.R., Taherkhani S., Talkhabi M. (2023). Alpha-ketoglutarate as a potent regulator for lifespan and healthspan: Evidences and perspectives. Exp. Gerontol..

[19] Sommer J.M., Newton A. (1988). Sequential regulation of developmental events during polar morphogenesis in Caulobacter crescentus: Assembly of pili on swarmer cells requires cell separation. J. Bacteriol..

[20] Guo L., Chen S., Ou L., Li S., Ye Z.N., Liu H.F. (2022). Disrupted Alpha-Ketoglutarate Homeostasis: Understanding Kidney Diseases from the View of Metabolism and Beyond. Diabetes Metab. Syndr. Obes..

[21] Tretter L., Adam-Vizi V. (2005). Alpha-ketoglutarate dehydrogenase: A target and generator of oxidative stress. Philos. Trans. R. Soc. B Biol. Sci..

[22] Ma X., Sun C., Ding X., Xu J., Zhang Y., Deng T., Wang Y., Yang H., Ding R., Li H. (2025). Mechanism analysis and targeted therapy of IDH gene mutation in glioma. Am. J. Cancer Res..

[23] MacDonald M.J., Brown L.J., Longacre M.J., Stoker S.W., Kendrick M.A., Hasan N.M. (2013). Knockdown of both mitochondrial isocitrate dehydrogenase enzymes in pancreatic beta cells inhibits insulin secretion. Biochim. Biophys. Acta.

[24] Ivanov S., Nano O., Hana C., Bonano-Rios A., Hussein A. (2024). Molecular Targeting of the Isocitrate Dehydrogenase Pathway and the Implications for Cancer Therapy. Int. J. Mol. Sci..

[25] Lu C., Ward P.S., Kapoor G.S., Rohle D., Turcan S., Abdel-Wahab O., Edwards C.R., Khanin R., Figueroa M.E., Melnick A. (2012). IDH mutation impairs histone demethylation and results in a block to cell differentiation. Nature.

[26] Zhou S., Wu H., Chen Y., Lv J., Chen S., Yu H., Shi T., Wang X., Xiao L. (2025). Lifting the veil on tumor metabolism: A GDH1-focused perspective. iScience.

[27] Di Conza G., Tsai C.H., Ho P.C. (2019). Fifty Shades of α-Ketoglutarate on Cellular Programming. Mol. Cell.

[28] Peyton K.J., Liu X.M., Yu Y., Yates B., Behnammanesh G., Durante W. (2018). Glutaminase-1 stimulates the proliferation, migration, and survival of human endothelial cells. Biochem. Pharmacol..

[29] Adam-Vizi V., Tretter L. (2013). The role of mitochondrial dehydrogenases in the generation of oxidative stress. Neurochem. Int..

[30] Millman S.E., Chaves-Perez A., Janaki-Raman S., Ho Y.J., Morris J.P.T., Narendra V., Chen C.C., Jackson B.T., Yashinskie J.J., Mezzadra R. (2025). α-Ketoglutarate dehydrogenase is a therapeutic vulnerability in acute myeloid leukemia. Blood.

[31] Wang Y., Guo Y.R., Liu K., Yin Z., Liu R., Xia Y., Tan L., Yang P., Lee J.H., Li X.J. (2017). KAT2A coupled with the alpha-KGDH complex acts as a histone H3 succinyltransferase. Nature.

[32] He L., Xu Z., Yao K., Wu G., Yin Y., Nyachoti C.M., Kim S.W. (2015). The Physiological Basis and Nutritional Function of Alpha-ketoglutarate. Curr. Protein Pept. Sci..

[33] Inigo M., Deja S., Burgess S.C. (2021). Ins and Outs of the TCA Cycle: The Central Role of Anaplerosis. Annu. Rev. Nutr..

[34] DeBerardinis R.J., Chandel N.S. (2016). Fundamentals of cancer metabolism. Sci. Adv..

[35] Guan R., Xue Z., Huang K., Zhao Y., He G., Dai Y., Liang M., Wen Y., Ye X., Liu P. (2025). α-Ketoglutarate Attenuates Oxidative Stress-Induced Neuronal Aging via Modulation of the mTOR Pathway. Pharmaceuticals.

[36] Liu L., Zhang W., Liu T., Tan Y., Chen C., Zhao J., Geng H., Ma C. (2023). The physiological metabolite α-ketoglutarate ameliorates osteoarthritis by regulating mitophagy and oxidative stress. Redox Biol..

[37] Yu H., Gan D., Luo Z., Yang Q., An D., Zhang H., Hu Y., Ma Z., Zeng Q., Xu D. (2024). α-Ketoglutarate improves cardiac insufficiency through NAD^+^-SIRT1 signaling-mediated mitophagy and ferroptosis in pressure overload-induced mice. Mol. Med..

[38] Milanovic M., Bekic M., Dokic J., Vucevic D., Colic M., Tomic S. (2024). Exogenous α-ketoglutarate Modulates Redox Metabolism and Functions of Human Dendritic Cells, Altering Their Capacity to Polarise T Cell Response. Int. J. Biol. Sci..

[39] Liu G., Lu J., Sun W., Jia G., Zhao H., Chen X., Wang J. (2024). Alpha-ketoglutaric acid attenuates oxidative stress and modulates mitochondrial dynamics and autophagy of spleen in a piglet model of lipopolysaccharide-induced sepsis. Free Radic. Biol. Med..

[40] Wang Y., Deng P., Liu Y., Wu Y., Chen Y., Guo Y., Zhang S., Zheng X., Zhou L., Liu W. (2020). Alpha-ketoglutarate ameliorates age-related osteoporosis via regulating histone methylations. Nat. Commun..

[41] Wu Z., Guan Y., Chen Q., Song R., Xie J., Zhang X., Wang Y., Chen Q., Chen X. (2025). Essential role of the metabolite α-ketoglutarate in bone tissue and bone-related diseases. Acta Biochim. Biophys. Sin..

[42] Chin R.M., Fu X., Pai M.Y., Vergnes L., Hwang H., Deng G., Diep S., Lomenick B., Meli V.S., Monsalve G.C. (2014). The metabolite α-ketoglutarate extends lifespan by inhibiting ATP synthase and TOR. Nature.

[43] Taylor T.D., Noguchi H., Totoki Y., Toyoda A., Kuroki Y., Dewar K., Lloyd C., Itoh T., Takeda T., Kim D.W. (2006). Human chromosome 11 DNA sequence and analysis including novel gene identification. Nature.

[44] Xu W., Yang H., Liu Y., Yang Y., Wang P., Kim S.H., Ito S., Yang C., Wang P., Xiao M.T. (2011). Oncometabolite 2-hydroxyglutarate is a competitive inhibitor of alpha-ketoglutarate-dependent dioxygenases. Cancer Cell.

[45] Srivastava A., Taly A.B., Gupta A., Murali T. (2015). Rehabilitation interventions to improve locomotor outcome in chronic stroke survivors: A prospective, repeated-measure study. Neurol. India.

[46] Xiao M., Yang H., Xu W., Ma S., Lin H., Zhu H., Liu L., Liu Y., Yang C., Xu Y. (2012). Inhibition of alpha-KG-dependent histone and DNA demethylases by fumarate and succinate that are accumulated in mutations of FH and SDH tumor suppressors. Genes. Dev..

[47] Lippi D. (2009). The transplant of the white man’s leg: A novel representation of Cosma and Damians miracle. Int. J. Immunopathol. Pharmacol..

[48] Liu J., Shangguan Y., Tang D., Dai Y. (2021). Histone succinylation and its function on the nucleosome. J. Cell. Mol. Med..

[49] Kotecha A., Perez-Martin E., Harvey Y., Zhang F., Ilca S.L., Fry E.E., Jackson B., Maree F., Scott K., Hecksel C.W. (2018). Chimeric O1K foot-and-mouth disease virus with SAT2 outer capsid as an FMD vaccine candidate. Sci. Rep..

[50] Martinez-Reyes I., Chandel N.S. (2020). Mitochondrial TCA cycle metabolites control physiology and disease. Nat. Commun..

[51] Weidinger S., Beck L.A., Bieber T., Kabashima K., Irvine A.D. (2018). Atopic dermatitis. Nat. Rev. Dis. Primers.

[52] Baghdanian A.H., Baghdanian A.A., Armetta A., Krastev M., Dechert T., Burke P., LeBedis C.A., Anderson S.W., Soto J.A. (2017). Effect of an Institutional Triaging Algorithm on the Use of Multidetector CT for Patients with Blunt Abdominopelvic Trauma over an 8-year Period. Radiology.

[53] Cutolo M., Campitiello R., Gotelli E., Soldano S. (2022). The Role of M1/M2 Macrophage Polarization in Rheumatoid Arthritis Synovitis. Front. Immunol..

[54] Liu S., Yang J., Wu Z. (2021). The Regulatory Role of α-Ketoglutarate Metabolism in Macrophages. Mediators Inflamm..

[55] Liu P.S., Wang H., Li X., Chao T., Teav T., Christen S., Di Conza G., Cheng W.C., Chou C.H., Vavakova M. (2017). α-ketoglutarate orchestrates macrophage activation through metabolic and epigenetic reprogramming. Nat. Immunol..

[56] Andersen J.V. (2025). The Glutamate/GABA-Glutamine Cycle: Insights, Updates, and Advances. J. Neurochem..

[57] Ugur B., Bao H., Stawarski M., Duraine L.R., Zuo Z., Lin Y.Q., Neely G.G., Macleod G.T., Chapman E.R., Bellen H.J. (2017). The Krebs Cycle Enzyme Isocitrate Dehydrogenase 3A Couples Mitochondrial Metabolism to Synaptic Transmission. Cell Rep..

[58] Lushnikova I., Kostiuchenko O., Kowalczyk M., Skibo G. (2023). mTOR/α-ketoglutarate signaling: Impact on brain cell homeostasis under ischemic conditions. Front. Cell. Neurosci..

[59] Calvo-Rodriguez M., Garcia-Rodriguez C., Villalobos C., Nunez L. (2020). Role of Toll Like Receptor 4 in Alzheimer’s Disease. Front. Immunol..

[60] Nguyen T.P., Caberlotto L., Morine M.J., Priami C. (2014). Network analysis of neurodegenerative disease highlights a role of Toll-like receptor signaling. BioMed Res. Int..

[61] Gao Y., Xiao Y., Hu Y., Yu L., Liu J., Zhang Z., Zhao T., Zhao S., Zhang L., Yang Y. (2025). α-Ketoglutarate alleviates the pathogenesis of lupus and inhibits the activation and differentiation of B cells by promoting the expression of CD39. Cell. Mol. Life Sci..

[62] Liu Y., Walter S., Stagi M., Cherny D., Letiembre M., Schulz-Schaeffer W., Heine H., Penke B., Neumann H., Fassbender K. (2005). LPS receptor (CD14): A receptor for phagocytosis of Alzheimer’s amyloid peptide. Brain.

[63] Reed-Geaghan E.G., Savage J.C., Hise A.G., Landreth G.E. (2009). CD14 and toll-like receptors 2 and 4 are required for fibrillar Abeta-stimulated microglial activation. J. Neurosci..

[64] Gambuzza M.E., Sofo V., Salmeri F.M., Soraci L., Marino S., Bramanti P. (2014). Toll-like receptors in Alzheimer’s disease: A therapeutic perspective. CNS Neurol. Disord. Drug Targets.

[65] Radziuk J. (2000). Insulin sensitivity and its measurement: Structural commonalities among the methods. J. Clin. Endocrinol. Metab..

[66] Sprigg W.A. (1996). Doctors watch the forecasts. Nature.

[67] Sarkar S., Chang C.I., Jean J., Wu M.J. (2025). TCA cycle-derived oncometabolites in cancer and the immune microenvironment. J. Biomed. Sci..

[68] Evans B., Griner E., Reproducibility Project: Cancer Biology (2015). Registered report: Oncometabolite 2-hydroxyglutarate is a competitive inhibitor of α-ketoglutarate-dependent dioxygenases. eLife.

[69] Abla H., Sollazzo M., Gasparre G., Iommarini L., Porcelli A.M. (2020). The multifaceted contribution of α-ketoglutarate to tumor progression: An opportunity to exploit?. Semin. Cell Dev. Biol..

[70] Calabrese C., Iommarini L., Kurelac I., Calvaruso M.A., Capristo M., Lollini P.L., Nanni P., Bergamini C., Nicoletti G., Giovanni C.D. (2013). Respiratory complex I is essential to induce a Warburg profile in mitochondria-defective tumor cells. Cancer Metab..

[71] Kurelac I., Iommarini L., Vatrinet R., Amato L.B., De Luise M., Leone G., Girolimetti G., Ganesh N.U., Bridgeman V.L., Ombrato L. (2019). Inducing cancer indolence by targeting mitochondrial Complex I is potentiated by blocking macrophage-mediated adaptive responses. Nat. Commun..

[72] Atlante S., Visintin A., Marini E., Savoia M., Dianzani C., Giorgis M., Surun D., Maione F., Schnutgen F., Farsetti A. (2018). alpha-ketoglutarate dehydrogenase inhibition counteracts breast cancer-associated lung metastasis. Cell Death Dis..

[73] Efimova E.V., Takahashi S., Shamsi N.A., Wu D., Labay E., Ulanovskaya O.A., Weichselbaum R.R., Kozmin S.A., Kron S.J. (2016). Linking Cancer Metabolism to DNA Repair and Accelerated Senescence. Mol. Cancer Res..

[74] Sun X., Zhu M.J. (2018). Butyrate Inhibits Indices of Colorectal Carcinogenesis via Enhancing alpha-Ketoglutarate-Dependent DNA Demethylation of Mismatch Repair Genes. Mol. Nutr. Food Res..

[75] Mi W., Xue Y., Yan H., Zhang Y., Cai X., Zhang S., He R., Li L., Zhu L., Xia X. (2026). α-Ketoglutarate dictates AMPK protein synthesis for energy sensing in human cancers. Nat. Chem. Biol..

[76] Wu F., Xie X., Li G., Bao D., Li H., Wu G., Lai Y., Xing Y., Ouyang P., Chen G. (2023). AKG induces cell apoptosis by inducing reactive oxygen species-mediated endoplasmic reticulum stress and by suppressing PI3K/AKT/mTOR-mediated autophagy in renal cell carcinoma. Environ. Toxicol..

[77] Zhou Y., Yu H., Cheng S., Chen Y., He L., Ren J., He X., Chen J., Zheng L., Li F. (2022). Glutamate dehydrogenase 1 mediated glutaminolysis sustains HCC cells survival under glucose deprivation. J. Cancer.

[78] Alhallaq A.S., Sultan N.S. (2025). Fueling Prostate Cancer: The Central Role of Glutamine/Glutamate Metabolic Reprogramming. Asian Pac. J. Cancer Prev..

[79] Dou H., Hao H., Zhao R., Li H., Wei F., Xu Y., Zheng D., Xie J., Li X. (2025). Dimethyl α-ketoglutarate ameliorates cisplatin-induced acute kidney injury by modulating mitophagy through the PINK1/Parkin pathway. Eur. J. Med. Res..

[80] Su Y., Wang T., Wu N., Li D., Fan X., Xu Z., Mishra S.K., Yang M. (2019). Alpha-ketoglutarate extends Drosophila lifespan by inhibiting mTOR and activating AMPK. Aging.

[81] Shahmirzadi A.A., Edgar D., Liao C.Y., Hsu Y.M., Lucanic M., Shahmirzadi A.A., Wiley C.D., Gan G., Kim D.E., Kasler H.G. (2020). Alpha-Ketoglutarate, an Endogenous Metabolite, Extends Lifespan and Compresses Morbidity in Aging Mice. Cell Metab..

[82] Demidenko O., Barardo D., Budovskii V., Finnemore R., Palmer F.R., Kennedy B.K., Budovskaya Y.V. (2021). Rejuvant^®^, a potential life-extending compound formulation with alpha-ketoglutarate and vitamins, conferred an average 8 year reduction in biological aging, after an average of 7 months of use, in the TruAge DNA methylation test. Aging.

[83] Stransky S., Graff S., Mao K., Huffman D.M., Milman S., Barzilai N., Sidoli S. (2026). Preliminary Evidence for Increased Histone Succinylation as a Potential Epigenetic Marker for Longevity. Aging Cell.

[84] Papadopoli D., Boulay K., Kazak L., Pollak M., Mallette F.A., Topisirovic I., Hulea L. (2019). mTOR as a central regulator of lifespan and aging. F1000Research.

[85] Kurhaluk N. (2024). Tricarboxylic Acid Cycle Intermediates and Individual Ageing. Biomolecules.

[86] Naeini F., Zeraattalab-Motlagh S., Rahimlou M., Ranjbar M., Hemmati A., Habibi S., Moradi S., Mohammadi H. (2024). Nutritional interventions in patients with burn injury: An umbrella review of systematic reviews and meta-analyses of randomised clinical trials. Br. J. Nutr..

[87] Stein J., Boehles H.J., Blumenstein I., Goeters C., Schulz R., Working Group for Developing the Guidelines for Parenteral Nutrition of The German Association for Nutritional Medicine (2009). Amino acids—Guidelines on Parenteral Nutrition, Chapter 4. Ger. Med. Sci..

[88] Coudray-Lucas C., Le Bever H., Cynober L., De Bandt J.P., Carsin H. (2000). Ornithine α-ketoglutarate improves wound healing in severe burn patients: A prospective randomized double-blind trial versus isonitrogenous controls. Crit. Care Med..

[89] Li Y., Bao X., Yang F., Tian J., Su W., Yin J., Yao K., Li T., Yin Y. (2022). Ornithine α-Ketoglutarate Alleviates Inflammation via Regulating Ileal Mucosa Microbiota and Metabolites in Enterotoxigenic *Escherichia coli*-Infected Pigs. Front. Nutr..

[90] Tian J., Yang F., Bao X., Jiang Q., Li Y., Yao K., Yin Y. (2023). Dietary Alpha-Ketoglutarate Supplementation Improves Bone Growth, Phosphorus Digestion, and Growth Performance in Piglets. Animals.

[91] Parker S.J., Encarnacion-Rosado J., Hollinshead K.E.R., Hollinshead D.M., Ash L.J., Rossi J.A.K., Lin E.Y., Sohn A.S.W., Philips M.R., Jones D.R. (2021). Spontaneous hydrolysis and spurious metabolic properties of α-ketoglutarate esters. Nat. Commun..

[92] Sun W., Han R., Xi H., Chen W., Li Y., Zhou Q., Li X., Huang K., Bontempo V., Gu X. (2025). Effects of Alpha-Ketoglutarate Supplementation on Growth Performance, Diarrhea Incidence, Plasma Amino Acid, and Nutrient Digestibility in Weaned Piglets. Animals.

[93] Niemiec T., Sikorska J., Harrison A., Szmidt M., Sawosz E., Wirth-Dzieciolowska E., Wilczak J., Pierzynowski S. (2011). Alpha-ketoglutarate stabilizes redox homeostasis and improves arterial elasticity in aged mice. J. Physiol. Pharmacol..

[94] Mangal J.L., Inamdar S., Suresh A.P., Jaggarapu M., Esrafili A., Ng N.D., Acharya A.P. (2022). Short term, low dose alpha-ketoglutarate based polymeric nanoparticles with methotrexate reverse rheumatoid arthritis symptoms in mice and modulate T helper cell responses. Biomater. Sci..

